# The Influence of Social Interaction on Cognitive Training for Schizophrenia

**DOI:** 10.3389/fnins.2012.00140

**Published:** 2012-09-28

**Authors:** Jennifer Louise Cook, Jennifer Black

**Affiliations:** ^1^Department of Psychiatry, University of CambridgeCambridge, UK; ^2^Technology transfer and administration, Cambridge EnterpriseCambridge, UK

In addition to the core features of the condition, individuals with schizophrenia typically exhibit deficits in cognitive functions (Barnett et al., 2010). Such impairments are known to be important determinants of functional outcome (Green et al., 2000; Harvey et al., 2003) and their magnitude is associated with the level of self-care, utilization of hospital services, and burden placed on caregivers (Davidson and Keefe, 1995; Sevy and Davidson, 1995; Martens and Addington, 2001).

The cognitive features of schizophrenia are poorly treated with antipsychotics (Liberman, 1994). Cognitive training offers a more promising option. Training interventions have been associated with cognitive improvements including information processing, verbal learning, and executive function (Medalia et al., 1998; Bellucci et al., 2003; McGurk et al., 2005; Sartory et al., 2005). The most recent meta-analysis found that, for individuals with schizophrenia, cognitive training improves cognitive function, psychosocial function, and psychiatric symptom severity (effect sizes of 0.45, 0.42, and 0.18 respectively; Wykes et al., 2011). Such findings highlight the promise of training programs in treating the cognitive symptoms of schizophrenia.

## Social Learning

Social Learning Theory (Bandura, 1977) postulates that much of human learning proceeds via observation and imitation, and predicts social learning to be more effective than non-social learning. Beneficial effects of social learning have been most commonly found in motor skill acquisition. For example, watching another person perform a task, such as a sequence of key presses, accelerates learning (Carroll and Bandura, 1990) by engaging the same learning mechanisms as direct practice (Bird and Heyes, 2005).

Beneficial effects of social learning are not limited to the motor domain, having also been noted in the language literature. Nine-month-old American babies, who have lost the ability to discriminate Mandarin sounds, are able to regain this ability after 12 sessions of face-to-face interaction with a Mandarin speaker, but not through observation of pre-recorded videos (Kuhl et al., 2003). Furthermore, educational programs that emphasize social interaction result in greater long-term improvements in academic achievement, social adjustment, and economic success than non-social programs (Ramey and Ramey, 2004; Heckman, 2006; Knudsen et al., 2006). These results have prompted the conclusion that social interaction plays an important role in various domains of learning throughout an individual’ s lifetime (Meltzoff et al., 2009).

## Does Social Interaction Improve Learning on Cognitive Training Tasks?

Cognitive training interventions are beginning to include social interaction in the training protocol (Hogarty, 2002; Vita et al., 2011; Tas et al., 2012). Although in its infancy, this field finds preliminary support for the proposition that combining cognitive training with social interaction may create optimal conditions for improving cognition in schizophrenia. However, the existing studies have typically used different methods of cognitive training for the social and non-social groups, hence conflating social learning and training type. The following section reviews a number of the extant studies and suggests directions for future research.

Vita et al. (2011) compared the effects of Integrated Psychological Therapy (IPT), computer-assisted cognitive remediation (CACR) therapy, and standard rehabilitation (Rehab) on symptomatological, neuropsychological, and functional outcome in schizophrenia. The IPT group practiced Cognitive Differentiation (classification of cards and formation of verbal concepts) and Social Perception (description and interpretation of social stimuli and discussions about social situations) for 24 weeks. Participants practiced for 45 min per session, twice a week, in groups of 8–10. The CACR group trained individually, for the same amount of time, on Cogpack (Marker Software^®^) which trains verbal memory, verbal fluency, psychomotor speed, executive function, working memory, and attention. The Rehab group completed group psychosocial sessions including art therapy, physical training, and occupational therapies for the same amount of time. Compared with the Rehab group, the IPT group, but not the CACR group, showed significantly greater improvement in mean processing speed and working memory scores. Given that IPT training involved two sessions per week of social interaction and CACR training did not involve social interaction, it may be that working with others facilitates learning and results in greater improvements than individual sessions. However, since the IPT and CACR groups differ in terms of both group size (group of 8–10 trainees versus individual training) and training type (ITP versus CACR) it is not clear from this study alone whether social interaction is the key factor in driving cognitive improvements.

Hogarty (2002) trained volunteers with schizophrenia for 2-years on either Cognitive Enhancement Therapy (CET) or Enhanced Supportive Therapy (EST). The CET group completed computer-based attention, memory, and problem solving exercises in pairs, and spent 1.5 h per week completing social cognitive exercises in groups of 6. The EST group received an educational intervention designed to improve illness self-management through coping strategies. EST was a manual-directed, office-based intervention supervised by an experimenter; social interaction tasks were not included in this training type. Processing speed, neurocognition (e.g. memory, language, cognitive flexibility), symptomatology, cognitive style (e.g. problems getting started, staying focused, and changing ideas), social cognition, and social adjustment were assessed at baseline and after training. The instruments used for assessment did not overlap with training instruments. After the 2-year training period, relative to baseline, improvements were significantly greater for the CET group compared to the EST group on all measures except symptomatology. Thus, group-based cognitive training (CET) resulted in improvements across the board relative to cognitive training in participant-experimenter pairs (EST). Given that social cognition and social adjustment were assessed using clinician-rated interviews about everyday social situations (e.g. relationships and employment) these results may translate well to real-life situations. Further work is necessary to establish whether this is true for neurocognitive improvements. As with the study by Vita et al. (2011) future work is required to isolate the beneficial features of CET – are the effects primarily driven by the size of the group or the nature of the tasks?

Tas et al. (2012) compared the effects of family assisted Social Cognition Interaction Therapy (f-SCIT) and Social Stimulation (SS) in participants with schizophrenia. F-SCIT focuses on emotion perception, Theory of Mind, and integration of learned skills into real-life. For f-SCIT, family members or friends were trained in social learning techniques and, once a week, acted as training-partners. The SS group met with a volunteer for 4 h every 3 weeks to complete activities such as visiting a café or taking part in art therapy. Training proceeded over a period of 14 weeks, with f-SCIT and SS receiving 18.6 and 16 h of training respectively. Social functioning, social cognition, and symptomatology assessments were carried out before and after training. The instruments used for assessment did not overlap with training instruments. The f-SCID group exhibited improvements in social functioning, social cognition, and symptoms compared to the SS group. Such effects may be due to facilitation of learning when the learning partner is a familiar other. However, further work is needed to elucidate whether this is indeed the case or whether the nature of the tasks and frequency of training played an important role in the success of the f-SCID program.

In sum, the extant studies suggest that social interaction may be an important factor in cognitive training for conditions like schizophrenia. Such a conclusion may have significant implications for therapy and should be further researched. Questions for future research include: is it social interaction *per se* that is responsible for improved learning rates? How important is the frequency of social interaction? And does the quality of social interaction matter?

## What Features of Social Interaction are Most Likely to be Beneficial to Learning?

Although the mechanisms that underpin social learning are still largely unknown (Heyes, 2011) a number of candidates have been suggested (Kuhl et al., 2003; Kuhl, 2007):

### Attention

Humans are particularly attention-grabbing stimuli. From birth, infants preferentially attend to face-like shapes rather than scrambled versions of faces or blank head-shaped stimuli (Morton and Johnson, 1991), and to upright rather than inverted point-light animations of biological motion (Simion et al., 2008). Infant preferential attention to faces and biological motion may contribute to the development of highly refined face (e.g. McKone et al., 2007) and human motion processing abilities (e.g. Cook et al., 2009: control group) that are evident in typical adults. A propensity to attend to, and superior processing abilities for, biological over non-biological stimuli may promote humans as a pertinent source of information.

### Arousal

Arousal has long been implicated in consolidation of learning; for instance retention of information is increased when stimulants are administered immediately following learning (Pare, 1961). Face-to-face teaching may comprise a highly arousing environment that promotes learning. In typically developing children, skin conductance response (SCR) amplitude, an index of arousal, is dynamically modulated during observation of facial expressions, with happy and sad faces being associated with greatest SCR amplitude (Skwerer et al., 2009). A plausible hypothesis is that emotional facial expressions, at key stages in teaching, promote arousal, and encourage consolidation of information.

### Adaptability of the learning resource

During teaching, tutors focus their eye-gaze on the information source and the infant’ s gaze tends to follow (Kuhl, 2007). In face-to-face situations tutors can monitor feedback from the infant and adapt their teaching accordingly. Video- and audio-tape based methods do not allow for such online adjustments. The “cues-filtered-out” approach suggests that the quality of an interaction is based upon the number of cues (e.g., non-verbal signals such as eyebrow flashes and verbal signals such as speech intonation) that a given media permits (Short et al., 1976; Daft and Lengel, 1984; Kock, 2004). The cue-rich nature of face-to-face interaction makes it an especially effective communication media. In addition to being rich sources of information, humans are also sophisticated reward delivery systems. Effective human tutors know to provide rewards at critical points in the learning process. Furthermore, for some individuals, social rewards (e.g. smiles) can be more motivating than non-social rewards (Kohls et al., 2009).

## Could a Machine Hi-Jack the Mechanisms of Action of Social Learning?

Recent discussion has highlighted the idea that cognitive training may be improved by using video-games as a means of delivering training (Sahakian et al., 2010; Sahakian, 2011). Video-games may hi-jack at least two of the three potential mechanisms of social learning: attention and arousal. However, at present it is unknown whether video-games are as attention-grabbing and arousal-inducing as real human face-to-face interaction.

In addition to being attention-grabbing and arousal-inducing the optimally successful method of cognitive training should adapt to the needs of the trainee and provide motivational rewards at critical moments in the learning process. Current methods of cognitive training are somewhat adaptive, presenting increasingly difficult problems as the participant’ s performance improves (e.g. Cogpack: Marker Software^®^). However, this is far from the adaptive abilities of a real human who can read the behavior of the trainee and use techniques, such as emphatic intonation of instructions, to promote learning. With rapid advances in the development of socially intelligent robots (Dautenhahn, 2007) the imminent future will reveal whether technology can meet these requirements.

## Summary and Implications for the Neurobiology of Social Learning

It is clear that developments in our understanding of the neurobiology of social learning may improve cognitive training. For example, elucidation of the psychological mechanisms of social learning may encourage the production of cognitive training protocols that emulate features of human tutors to achieve optimal learning. Reciprocally, developments in cognitive training may benefit our understanding of the neurobiology of social learning. An intriguing question concerns whether social learning proceeds via domain-specific or domain-general mechanisms. By definition non-human teaching devices cannot operate via social-specific learning mechanisms. Thus, the view that social learning proceeds via domain-general learning mechanisms, would be supported by the existence of non-human devices that match human teaching successes. Analyses of the development of successful cognitive training regimes may also elucidate those features of learning protocols that are critical to learning and those which are extraneous. Such findings may highlight important, and underexplored, features of the neurobiology of social learning.
